# Editorial: Highlights in General Cardiovascular Medicine: 2021

**DOI:** 10.3389/fcvm.2022.904239

**Published:** 2022-05-26

**Authors:** Lijun Wang, Longlu Pan, Pietro Enea Lazzerini, Junjie Xiao

**Affiliations:** ^1^School of Medicine, Affiliated Nantong Hospital of Shanghai University (The Sixth People's Hospital of Nantong), Institute of Geriatrics, Shanghai University, Nantong, China; ^2^Cardiac Regeneration and Ageing Lab, Shanghai Engineering Research Center of Organ Repair, School of Life Science, Institute of Cardiovascular Sciences, Shanghai University, Shanghai, China; ^3^Department of Medical Sciences, Surgery, and Neurosciences, University of Siena, Siena, Italy

**Keywords:** Frontiers in Cardiovascular Medicine, General Cardiovascular Medicine Section, best paper, 2021, editorial

This editorial features the selection of highlights articles published in Frontiers in Cardiovascular Medicine (General Cardiovascular Medicine Section) in 2021. Our criteria for selection include: whether the topic addressed in the study is highly relevant and timely importance in cardiovascular medicine, show the quality and breadth of the papers published in this section, as well as geographical variety; whether the article is often viewed, downloaded, and cited; and whether the article is well-written and has good readability. Following are the highlight papers in the General Cardiovascular Medicine Section of the year 2021 (Table 1).

**Table 1:** Metrics (on April 21st 2022) of the highlight's articles published in Frontiers in Cardiovascular Medicine, General Cardiovascular Medicine section, in 2021.

**Title**	**First author, Country**	**Views**	**Downloads^#^**
Empagliflozin in patients with heart failure: a systematic review and meta-analysis of randomized controlled trials	Deng Pan, China	4,284	774
Does prenatal exposure to CNS stimulants increase the risk of cardiovascular disease in adult offspring?	Boyd R. Rorabaugh, United States	1,767	772
The long-term impact of bariatric surgery on development of atrial fibrillation and cardiovascular events in obese patients: an historical cohort study	Hongtao Yuan, China	1,971	671
Quality assessment of published systematic reviews in high impact cardiology journals: revisiting the evidence pyramid	Abdelrahman I. Abushouk, United States	3,710	649
Routine oxygen therapy does not improve health-related quality of life in patients with acute myocardial infarction—insights from the randomized DETO2X-AMI Trial	Robin Hofmann, Sweden	3,099	635
Impact of the 2019 novel coronavirus disease pandemic on the performance of a cardiovascular department in a non-epidemic center in Beijing, China	Jing Nan, China	1,799	554
High-normal serum magnesium and hypermagnesemia are associated with increased 30-day in-hospital mortality: a retrospective cohort study	Liao Tan, China	1,781	468
From clinical clues to final diagnosis: the return of detective work to clinical medicine in cardiac amyloidosis	Hani Sabbour, United arab emirates	3,004	428
Association of N-terminal pro-brain natriuretic peptide with volume status and cardiac function in hemodialysis patients	Yaqiong Wang, China	1,887	394
Energy drink-associated electrophysiological and ischemic abnormalities: a narrative review	Diana X. Cao, United States	2,724	392

# *The order of appearance according to the number of downloads (on April 21, 2022)*.

## Highlights in General Cardiovascular Medicine: 2021

– *Empagliflozin in Patients With Heart Failure: A Systematic Review and Meta-Analysis of Randomized Controlled Trials* (Pan et al.).

The prevalence of heart failure (HF) is increasing and leads to high mortality (1). This systemic review evaluated the effect of empagliflozin in patients with HF. In this paper, the authors provided the first systematic review and meta-analysis that focused on the effect and potential mechanism of empagliflozin in patients with HF. This study provided information for clinicians that empagliflozin can significantly reduce the composite of cardiovascular death or hospitalization for worsening heart failure, as well as indicating that further long-term randomized controlled trials, with uniformed doses and durations of follow-up to system analysis of the effect of empagliflozin in HF patients, are required.

– *Does Prenatal Exposure to CNS Stimulants Increase the Risk of Cardiovascular Disease in Adult Offspring*? (Rorabaugh).

The long-term negative effects of prenatal exposure to central nervous system (CNS) stimulants on adult offspring's cardiovascular system have received much attention recently. This review summarized the current knowledge of the impact of prenatal exposure to cocaine, methamphetamine, nicotine, and caffeine on adult cardiovascular function. This study suggested that exposure to CNS stimulants during the gestational period can negatively impact the adult heart later in life. The observations in this paper provided important implications for clinicians and researchers that pregnant women should be more strictly recommended to avoid CNS stimulant exposure.

– *The Long-Term Impact of Bariatric Surgery on Development of Atrial Fibrillation and Cardiovascular Events in Obese Patients: An Historical Cohort Study* (Yuan et al.).

The prevalence of obesity is increasing worldwide and is highly associated with an increased incidence of metabolic diseases. This study demonstrated that Roux-en-Y gastric bypass (RYGB) surgery in patients with obesity improves the metabolic profile and was associated with lower cardiovascular events, while did not demonstrate a benefit for atrial fibrillation prevention in the young population. This paper provided recommendations for clinical researchers that longer time follow-up is needed to evaluate the benefit of bariatric surgery on AF.

– *Quality Assessment of Published Systematic Reviews in High Impact Cardiology Journals: Revisiting the Evidence Pyramid* (Abushouk et al.).

A growing number of systematic reviews and meta-analyses are published in recent years and used as sources of evidence in clinical and animal cardiology guidelines (2–4). However, concerns have been raised regarding the expertise, quality, and methodological standards of many systemic reviews (5, 6). In this study, the authors performed a comprehensive quality assessment of published systematic reviews in high-impact, general cardiology/cardiovascular medicine journals. This study represents the first comprehensive analysis of the methodological characteristics and gaps of systematic reviews in the cardiology literature. This study also provided recommendations for researchers, clinicians, journal editors, and peer reviewers, as well as policymakers to improve the quality of systematic reviews in cardiovascular medicine.

– *Routine Oxygen Therapy Does Not Improve Health-Related Quality of Life in Patients With Acute Myocardial Infarction-Insights From the Randomized DETO2X-AMI Trial* (Hofmann et al.).

Oxygen therapy has been used in patients with acute myocardial infarction (MI) worldwide. Limited studies have been focusing on oxygen therapy's effects on health-related quality of life (HRQoL). This study investigated the impact of routine oxygen supplementation on HRQoL 6–10 weeks after hospitalization with acute MI and suggested that routine oxygen therapy provided to acute MI patients did not improve HRQoL up to 1 year after MI occurrence, regardless of MI subtype. This study also provided important evidence that oxygen therapy should be used more carefully in patients with hypoxemia.

– *Impact of the 2019 Novel Coronavirus Disease Pandemic on the Performance of a Cardiovascular Department in a Non-epidemic Center in Beijing* (Nan et al.).

The 2019 novel coronavirus disease (COVID-19) has become a global pandemic since 2020. This research paper investigated the impact of the COVID-19 pandemic on the normal performance of a cardiovascular department in a large non-epidemic hub center in Beijing, China. This study indicated that the hub-and-spoke model could be effective in limiting the collateral damage for patients affected by cardiovascular diseases when the medical system was stressed by disasters, such as the COVID-19 pandemic.

– *High-Normal Serum Magnesium and Hypermagnesemia Are Associated With Increased 30-Day In-Hospital Mortality: A Retrospective Cohort Study* (Tan et al.).

Serum magnesium level homeostasis is important for cardio-physiological regulation. The association between serum magnesium levels and acute cardiovascular events remains to be studied. This study investigated the association of admission serum magnesium level with all-cause in-hospital mortality in critically ill patients with acute myocardial infarction (AMI). High-normal serum magnesium and hypermagnesemia are both independent predictors of 30-day in-hospital mortality in ICU patients with AMI. Serum magnesium detection in the patient is simple and low cost. This study indicated that routinely monitoring serum magnesium levels might be acted as a potential predictor for AMI.

– *From Clinical Clues to Final Diagnosis: The Return of Detective Work to Clinical Medicine in Cardiac Amyloidosis* (Sabbour et al.).

Cardiac amyloidosis is characterized by extracellular protein fibril deposition in the myocardium. Limited evidence and guideline recommendations are provided for the diagnosis and management of cardiac amyloidosis (7). In this study, the authors provided a guide on how to recognize the often-overlooked presentations of this disease in clinical practice, and diagnostic algorithms as well as case examples are presented.

– *Association of N-Terminal Pro-brain Natriuretic Peptide With Volume Status and Cardiac Function in Hemodialysis Patients* (Wang et al.).

This study enrolled patients who had been receiving hemodialysis for >3 months and analyzed the relationship between N-terminal-pro-brain natriuretic peptide (NT-pro BNP) level and body fluid status in dialysis patients with reduced cardiac ejection function (EF). This paper suggested that NT-pro BNP could be a predictive factor for volume overload in hemodialysis patients with or without EF declines.

– *Energy Drink-Associated Electrophysiological and Ischemic Abnormalities: A Narrative Review* (Cao et al.).

An increasing number of cardiovascular adverse effects, emergency room visits, and deaths have been linked to the consumption of energy drinks (8, 9). This study comprehensively reviewed the correlation between energy drink consumption and electrophysiological and ischemic adverse effects. This paper also suggested that energy drink use should be considered as part of the differential diagnosis in appropriate patients presenting with electrocardiographic changes.

## Author Contributions

JX and PL had the idea for the article and revised the article. LW and LP drafted the article. All authors contributed to the article and approved the submitted version.

## Funding

This work was supported by the grants from National Key Research and Development Project (2018YFE0113500 to JX), National Natural Science Foundation of China (82020108002 to JX), Innovation Program of Shanghai Municipal Education Commission (2017-01-07-00-09-E00042 to JX), the grant from Science and Technology Commission of Shanghai Municipality (21XD1421300 and 20DZ2255400 to JX), Natural Science Foundation of Shanghai (19ZR1474100 to LW), and the “Dawn” Program of Shanghai Education Commission (19SG34 to JX).

## Conflict of Interest

The authors declare that the research was conducted in the absence of any commercial or financial relationships that could be construed as a potential conflict of interest.

## Publisher's Note

All claims expressed in this article are solely those of the authors and do not necessarily represent those of their affiliated organizations, or those of the publisher, the editors and the reviewers. Any product that may be evaluated in this article, or claim that may be made by its manufacturer, is not guaranteed or endorsed by the publisher.
